# Systematic Review of Utilized Ports in Laparoscopic Cholecystectomy: Pushing the Boundaries

**DOI:** 10.1155/2024/9961528

**Published:** 2024-05-23

**Authors:** Shadi A. Alshammary, Dhuha N. Boumarah

**Affiliations:** Department of Surgery, King Fahd Hospital of the University, College of Medicine, Imam Abdulrahman Bin Faisal University, Dammam, Saudi Arabia

## Abstract

**Introduction:**

Surgical procedures have undergone a paradigm shift in the last 3 decades, with minimally invasive surgery becoming standard of care for a number of surgeries, including the treatment of benign gallbladder diseases. By providing a thorough and impartial summary of the earlier published systematic reviews, the current systematic review is the first to present comparison results. This review illustrates the data of intraoperative and postoperative results of each laparoscopic cholecystectomy technique.

**Materials and Methods:**

The Preferred Reporting Items for Systematic Reviews and Meta-Analyses (PRISMA) guideline was meticulously followed to conduct the present systematic review. MEDLINE (via PubMed), Cochrane Database of Systematic Reviews, and Web of Science were searched for eligible publications, and a total of 14 systematic reviews were included. A newly developed extraction table was utilized to obtain the predefined parameters from eligible systematic reviews, including operative time, conversion rate, estimated blood loss, bile leak, length of hospital stay, postoperative pain, and cosmetic results. All statistical analyses were conducted using Statistical Package for the Social Sciences (SPSS) software, version 26.0. The analysis of dichotomous results was summarized using relative risks and 95% confidence intervals (95% CI), and continuous results were summarized using mean differences and 95% CIs. The proportions were compared using a single proportion *z*-test.

**Results:**

The analysis of our primary and secondary outcomes revealed a statistically significant improvement in aesthetic results after single-incision laparoscopic cholecystectomy (SILC) in comparison to the multiport approach of laparoscopic cholecystectomy. This, however, is accompanied by extended operative timing and subsequently, prolonged exposure to anesthesia.

**Conclusion:**

Patients should be carefully selected for SILC to minimize technical difficulties and prevent complications both intraoperatively and shortly after the procedure. This trial is registered with CRD42023392037.

## 1. Introduction

The past decade has witnessed a paradigm shift in surgical interventions, with minimally invasive surgery being superior to the traditional open approach for several conditions, including the management of benign gallbladder diseases [1]. Cholelithiasis, for instance, is a frequently encountered clinical condition, with an overall prevalence estimated to reach up to 27% among the general population. Despite the fact that the vast majority of patients remain asymptomatic throughout their lifetime, around 1–4% of affected individuals manifest clinically significant symptoms, which might mandate a surgical intervention [2]. The widespread acceptance and popularity of laparoscopic cholecystectomy, in fact, can be explained by the significant reduction in associated morbidity and faster recovery to daily activities, in comparison to the classic open approach [3]. The first laparoscopic cholecystectomy was performed by Erich Muhe in 1985 [4]. Technical aspects have evolved tremendously since then to include a variety of laparoscopic options with a lower number and size of ports in an attempt to minimize invasiveness. Three-port laparoscopic cholecystectomy and single-incision laparoscopic cholecystectomy (SILC) were introduced as alternatives to the classic four-port laparoscopic cholecystectomy in an effort to augment the benefit of minimally invasive techniques [5, 6]. Given the fact that pain postoperatively represents the most frequent complaint following laparoscopic cholecystectomy, reduction of port number or size might enhance the patient experience and result in greater patient satisfaction overall [7]. Previous studies have briefly addressed the differences between the three approaches of laparoscopic cholecystectomy (namely, traditional four-port and three-port laparoscopic cholecystectomy and SILC) in terms of feasibility and safety. However, there is still a paucity of evidence in regard to the optimal technique for utilization and controversy regarding the overall clinical outcomes. The current systematic review is the first to provide comparative outcomes by summarizing the previously published systematic reviews in a comprehensive and objective pattern. It aims to illustrate evidence regarding the intraoperative and postoperative outcomes of each laparoscopic cholecystectomy approach, as most of previous publications have only briefly touched upon one or two of them.

## 2. Materials and Methods

The current systematic review was conducted in accordance with the Preferred Reporting Items for Systematic Reviews and Meta-Analyses (PRISMA) guidelines [8]. A protocol was developed prior to the extraction and analysis of data to guide the various steps of this systematic review and was registered in the PROSPERO database, with the registration number CRD42023392037.

### 2.1. Search Strategy

The literature was systematically reviewed and the following electronic databases: MEDLINE (via PubMed), Cochrane Database of Systematic Reviews, and Web of Science were searched for eligible publications until January 15, 2023. Systematic reviews addressing the clinical outcomes of different approaches of laparoscopic cholecystectomy (specifically, comparing conventional laparoscopic cholecystectomy with SILC or three-port laparoscopic cholecystectomy) were included, only if limited to randomized controlled trials (RCTs). Other criteria that were considered prior to inclusion are the language of publications as only articles published in English language were eligible for inclusion. No publication year restriction was applied and all systematic reviews fulfilling the inclusion criteria were reviewed for extraction. Bibliographic reference lists of included systematic reviews were screened for eligible articles. Articles were excluded if written in a non-English language, not a systematic review, addressing the minimal invasive approach of other surgical intervention than laparoscopic cholecystectomy, addressing the open approach of cholecystectomy or systematic review but not limited to RCTs. The following MeSH terms were used for literature search: “laparoscopic cholecystectomy” or “minimally invasive cholecystectomy” combined with one of the following “SILC” or “single-incision” or “three-port” or “reduced-port” or “conventional” or “classic” or “comparison,” in combination with the term “systematic review.”

### 2.2. Study Selection

The two authors independently screened the title and abstract of the initially identified articles and irrelevant articles were excluded accordingly. Full-text papers of articles fulfilling the inclusion criteria were retrieved and assessed for eligibility against the predetermined inclusion criteria by both authors.

### 2.3. Data Extraction and Quality Assessment

The assessed outcomes were divided into intraoperative and postoperative parameters. Intraoperative outcomes included operative time, estimated blood loss, bile duct injury, and bile leak, along with the rate of conversion to open surgery. For postoperative outcomes assessment, on the other hand, the following variables were extracted: pain and requirement of analgesia, length of hospital stay, and cosmetic results. A newly developed extraction table was utilized to obtain the predefined parameters from eligible systematic reviews, including the following: (1) year of publication, (2) compared surgical technique, (3) number of included RCTs and patients, (4) operative time, (5) conversion rate, (6) estimated blood loss, (7) bile leak, (8) length of hospital stay, (9) postoperative pain, and (10) cosmetic results. One author extracted the data and the other one reviewed them for accuracy. The methodological quality of included systematic reviews was then assessed by one reviewer and verified by another one, using the AMSTAR-2 tool, which consists of 16 domains for critical appraisal of systematic reviews [9].

### 2.4. Statistical Analysis

All statistical analyses were conducted using Statistical Package for the Social Sciences (SPSS) software, version 26.0. The analysis of dichotomous results was summarized using relative risks and 95% confidence intervals (95% CI), and continuous results were summarized using mean differences and 95% CIs. One-proportion *Z*-test was utilized to compare the proportions, with *p* value defined as statistically significant if <0.05.

## 3. Results

**Figure 1:**
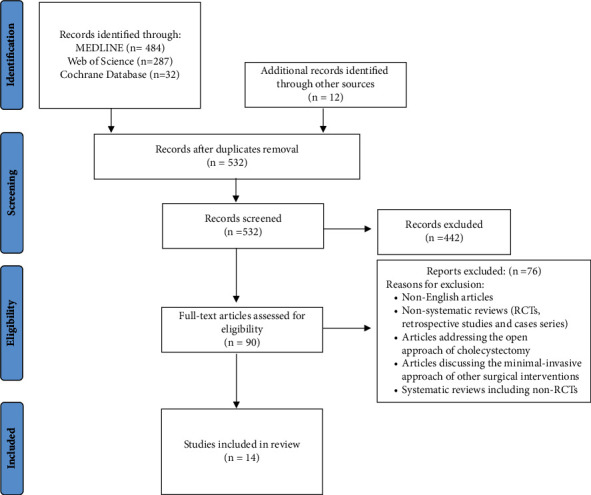
PRISMA flow diagram summarizing the literature search process and study selection.

The initial search of databases yielded 803 records, and 12 additional records were identified through retrieval of references. After duplicate removal, 532 articles were screened for inclusion by titles and abstracts. 442 publications were excluded as their objectives were considered irrelevant for our systematic review. The full text of 90 articles was reviewed to assess eligibility prior to inclusion. Systematic reviews including non-RCTs were excluded as an attempt to reduce inevitable bias. We included 14 systematic reviews fulfilling our previously identified inclusion criteria.  Figure 1 represents details of the search strategy according to PRISMA guidelines. The included systematic reviews were published between 2012 and 2022, with a total number of 271 RCTs. Out of the included 14 articles, four were conducted in the United Kingdom, three were carried out in Italy, two in China, and the remaining were performed in the Netherlands (*n* = 1), Indonesia (*n* = 1), Switzerland (*n* = 1), Croatia (*n* = 1), and the United States (*n* = 1). The vast majority of systematic reviews compared single-incision laparoscopic cholecystectomy with multiport approach of cholecystectomy, and only a single systematic review specified three-port and four-port approaches in their comparison.  Table 1 summarizes the characteristics of included systematic reviews along with highlighting results of interest. As quality assessment of included publications was carried out utilizing the AMSTAR-2 tool for critical appraisal, two reviews were found to have high quality, one with moderate quality, 5 were classified as low quality, and 6 with critically low quality.  Table 2 illustrates the methodological assessment result of included studies.

### 3.1. Primary Outcomes

#### 3.1.1. Experienced Postoperative Pain

Data regarding postoperative pain experienced by patients and the requirement of analgesia were reported by all included systematic reviews, 6 publications (42.9%) reported lower rates of postoperative pain in SILC and three-port laparoscopic cholecystectomy in comparison to the classic four-port approach [6, 10–14], whereas 8 reviews (57.1%) showed no difference between the different groups (*p*=0.59, *z* = 0.53) [4, 15–21]. Significant heterogeneity of data was observed as the assessment of pain was not generalized among studies; some articles utilized the visual analog scale for pain assessment and other studies, however, kept pain evaluation subjective to patients. Other source of heterogeneity is the duration of postoperative evaluation as it was not identified in some studies and was limited to 6 hours postoperatively in others.

#### 3.1.2. Length of Hospital Stay

Length of hospital stay was investigated in all 14 included systematic reviews, only 1 study (7.1%) reported a reduction in length of hospital stay in three-port laparoscopic cholecystectomy in comparison to the four-port approach [6]. Thirteen publications (92.9%) showed no significant difference between the available approaches in terms of hospital stay duration (*p*=0.001, *z* = 3.21) [4, 10–21].

#### 3.1.3. Cosmesis

Twelve articles (85.7%) contributed to the analysis of cosmetic results and two articles (14.3%) did not touch on this aspect [11, 21]. Out of the 12 systematic reviews reporting the variation in cosmesis between the 3 approaches, 10 publications (71.4%) illustrated evidence of better cosmetic results with SILC in comparison to four-port laparoscopic cholecystectomy (*p* < 0.0001, *z* = 6.47) [4, 10, 12–18, 20], and only 2 studies (14.3%) demonstrated no difference between any of the approaches [6, 19].

### 3.2. Secondary Outcomes

#### 3.2.1. Operative Time

The calculation of the operative time variability was obtained after the inclusion of 14 systematic reviews, no difference in operative time was observed between three- and four-port laparoscopic cholecystectomy [6]. However, a significant difference was seen between SILC and multiport laparoscopic cholecystectomy, with longer time required to perform SILC (*p*=0.001, *z* = 3.21) (MD: 14.08 minutes, SD: 4.29), the range reported by included studies varied between 10 and 23 minutes [4, 10–21].

#### 3.2.2. Conversion Rate

Conversion rate, as defined by the requirement to convert a laparoscopic approach to open cholecystectomy, was reported in all 14 studies. The percentage of conversion as defined by the addition of 1 or 2 ports was not included in the analysis of this variable. Only one systematic review (7.1%) showed a higher rate of conversion in association with SILC compared to multiport laparoscopic cholecystectomy [6]. 13 articles (92.9%) illustrated no difference in the rate of conversion from laparoscopic to open approach between the three different laparoscopic approaches (*p*=0.001, *z* = 3.21) [4, 10–21].

#### 3.2.3. Estimated Blood Loss

The estimated volume of blood loss was reported in 9 publications [4, 6, 10, 12–15, 20, 21]. 6 systematic reviews demonstrated no difference in blood loss estimate between the three approaches [6, 10, 12, 14, 15, 21]. Only 3 studies showed a higher rate of intraoperative blood loss during SILC [4, 13, 20].

#### 3.2.4. Bile Leak/BDI

Out of the 14 included studies, the observation of bile leakage and bile duct injury was available in 13 systematic reviews (92.9%), as one of the included publications (7.1%) did not address this aspect [19]. 2 studies showed a more frequent occurrence of bile duct injury and bile leak in SILC in comparison to multiport laparoscopic cholecystectomy [11, 13]. 11 articles, on the other hand, reported equal incidence between the 3 approaches (*p* < 0.0001, *z* = 4.06) [4, 6, 10, 12, 14–18, 20, 21].

## 4. Discussion

The current systematic review is the first to summarize the evidence available from systematic reviews addressing the different laparoscopic techniques of cholecystectomy and comparing their intraoperative and postoperative clinical outcomes. Since the introduction of SILC by Navarra et al. in 1997, extensive efforts have been made to prove its safety and feasibility to replace the conventional method of laparoscopic cholecystectomy [22]. The analysis of our primary and secondary outcomes revealed a statistically significant improvement in aesthetic results after SILC in comparison to the multiport approach of laparoscopic cholecystectomy. This, however, is accompanied by extended operative timing and subsequently prolonged exposure to anesthesia. Therefore, careful selection of candidates undergoing SILC should be present to minimize technical difficulties and prevent related complications both intraoperatively and shortly after the procedure. Due to the compromised intraoperative visualization in obese individuals and the difficulty in identifying biliary structures secondary to the presence of fatty tissues, SILC is better to be limited to patients with a BMI of 30 or less to minimize the risk of complications [23]. It is essential to note that the majority of included systematic reviews have excluded obese population from their analysis, which limits generalizability of our findings among them.

Previous surgeries involving the abdomen and signs of acute cholecystitis were identified as factors predicting failure of SILC, with a success rate limited to 59% in association with acute cholecystitis, as suggested by Antoniou et al. in his systematic review addressing the limitations of the single-incision approach [24]. Furthermore, the American Society of Anesthesiologists (ASA) classification might be utilized as a tool to exclude patients predisposed to risk of prolonged anesthesia, as those with a score of 3 and more were excluded from some of the included systematic reviews allocated in our study [16].

The associated increase in length of operative time can be partly explained by the surgeon learning curve as applied to any surgical procedure. Supporting this postulation is the observation of Chow et al., who spent around 202 minutes to perform their first SILC and, in contrast, the last case required 75 minutes [25]. Moreover, a Chinese study conducted by Xiao et al. in 2010, comparing SILC and conventional laparoscopic cholecystectomy, suggested that the operative time of SILC in their hands reached the plateau with experience and became comparable to the required time of the conventional technique reaching around 52 minutes [26, 27]. Given these considerations, it is evident that the relatively lower experience is building a barrier to optimal implementation of the single-incision technique of laparoscopic cholecystectomy. An additional aspect for comparison that was underinvestigated by the majority of the previously published literature is the incidence of port-site hernia following SILC compared to conventional intervention. One of the included systematic reviews conducted by Lyu et al. illustrated an increased rate of incisional hernia following SILC [15]. However, the review by Rudiman and Hanafi reported a similar rate of incisional hernia occurrence among both interventions, namely, SILC and conventional approach [10]. Therefore, this specific complication needs to be adequately investigated in future prospective studies.

SILC seems to be superior to the three- and four-port laparoscopic cholecystectomy in regard to aesthetic outcomes seen postoperatively with comparable safety profiles. Although the present review is the first to demonstrate clear generalized evidence regarding the differences between the three laparoscopic approaches of cholecystectomy, the heterogeneity of included studies and the variation in surgical preferences might be one of the limitations. Differences in the interpretation of pain and cosmesis among allocated studies might also affect outcome evaluation. Future research should focus on providing scientific data with higher level of evidence conducted and reported in a more comprehensive manner to establish and formulate an accurate background about the single-incision approach of cholecystectomy.

## 5. Conclusion

The present systematic review provides comparison between the three different approaches of laparoscopic cholecystectomy and sheds light on the applicability and potential of SILC. Three-port cholecystectomy showed similar outcomes, safety, and applicability comparing to the traditional four-port cholecystectomy. Nevertheless, based on the findings provided from the current review, single-incision laparoscopic cholecystectomy is a feasible alternative to the traditional multiport approach with better aesthetic results but longer operative duration and learning curve. Wise selection of patients remains crucial to ensure the provision of a safe and time-effective management. For more compelling findings regarding the optimal approach and the precise indications of SILC, further high-quality evidence is required to draw a definitive, more detailed conclusion.

## Figures and Tables

**Table 1:** Summary of included systematic reviews illustrating the investigated intraoperative and postoperative outcomes.

Serial no.	Author(s)/year	Compared techniques	Included studies	Number of patients	Operative time	Conversion rate	Estimated blood loss	Bile leak/BDI	Length of stay	Postoperative pain	Cosmetic results
1	Nip et al. [6] (2022)	Three- and four-port techniques	18 RCTs	2,085 patients	No difference	No difference	No difference	No difference	Lower with three-port approach	Lower with three-port approach	No difference
2	Rudiman and Hanafi [10] (2022)	SILC and CLC	37 RCTs	4,521 patients	SILC is 10 minutes longer	No difference	No difference	No difference	No difference	Less with SILC 6 hours postoperatively	SILC offers a better cosmesis
3	Pereira and Gururaj [11] (2022)	SILC and CLC	14 RCTs	1,762 patients	SILC is 19 minutes longer	No difference	Not reported	Higher rates with SILC	No difference	Less with SILC 4–6 hours postoperatively	Not reported
4	Lyu et al. [12] (2019)	SILC and CLC	48 RCTs	5,794 patients	SILC is 15 minutes longer	No difference	No difference	No difference	Not reported	No difference	SILC offers a better cosmesis
5	Haueter et al. [13] (2017)	SILC and CLC	37 RCTs	3,051patients	SILC is 14 minutes longer	No difference	No difference	No difference	No difference	Less with SILC 12 hours postoperatively	SILC offers a better cosmesis
6	Evers et al. [14] (2017)	SILC and CLC	9 RCTs	860 patients	SILC is 23 minutes longer	No difference	Higher rates with SILC	Higher rates with SILC	No difference	Less with SILC	SILC offers a better cosmesis
7	Lirici et al. [15] (2016)	SILC and CLC	17 RCTs	1,293 patients	SILC is 20 minutes longer	No difference	Not reported	No difference	No difference	No difference	SILC offers a better cosmesis
8	Milas et al. [4] (2014)	SILC and CLC	30 RCTs	2,411 patients	SILC is 12 minutes longer	No difference	Higher rates with SILC	No difference	No difference	No difference	SILC offers a better cosmesis
9	Zehetner et al. [16] (2013)	SILC and CLC	9 RCTs	695 patients	SILC is 12 minutes longer	No difference	Not reported	No difference	No difference	No difference	SILC offers a better cosmesis
10	Arezzo et al. [17] (2013)	SILC and CLC	12 RCT	996 patients	SILC is 11 minutes longer	No difference	No difference	No difference	No difference	Less with SILC	SILC offers a better cosmesis
11	Wang et al. [18] (2012)	SILC and CLC	9 RCTs	652 patients	SILC is 10 minutes longer	Higher rates with SILC	Not reported	No difference	No difference	No difference	SILC offers a better cosmesis
12	Sajid et al. [19] (2012)	SILC and CLC	11 RCTs	1,858 patients	SILC is 10 minutes longer	No difference	Not reported	Not reported	No difference	No difference	No difference
13	Trastulli et al. [20] (2012)	SILC and CLC	13 RCTs	923 patients	SILC is 16 minutes longer	No difference	Higher rates with SILC	No difference	No difference	No difference	SILC offers a better cosmesis
14	Markar et al. [21] (2012)	SILC and CLC	7 RCTs	335 patients	SILC is 11 minutes longer	No difference	No difference	No difference	No difference	No difference	Not reported

**Table 2:** Quality assessment of included systematic reviews.

Study	AMSTAR-2 items																Quality rating
	Item 1	Item 2	Item 3	Item 4	Item 5	Item 6	Item 7	Item 8	Item 9	Item 10	Item 11	Item 12	Item 13	Item 14	Item 15	Item 16	
Nip et al.	Partial yes	Yes	Yes	Yes	Yes	Yes	Yes	Yes	Yes	Yes	Yes	Yes	Yes	Yes	Yes	Yes	High quality
Rudiman et al.	Yes	Yes	Yes	Yes	Yes	Yes	Yes	Yes	Yes	No	Yes	Partial yes	No	No	Yes	No	Low quality
Pereira et al.	Partial yes	No	Yes	Yes	Yes	Yes	Yes	Yes	Yes	Yes	Yes	No	No	Yes	No	Yes	Critically low quality
Lyu et al.	No	No	Yes	Yes	No	Yes	No	Yes	Yes	Yes	Yes	Yes	Yes	Yes	Yes	Yes	Critically low quality
Haueter et al.	Yes	Yes	Yes	Yes	Yes	Yes	Yes	Yes	Yes	No	Yes	Yes	Yes	Yes	Yes	No	Moderate quality
Evers et al.	Yes	No	Yes	Yes	Yes	Yes	Yes	Yes	Yes	No	Yes	Yes	Yes	Yes	Yes	Yes	Low quality
Lirici et al.	Yes	No	Yes	Yes	No	No	Yes	Yes	No	Yes	Yes	N/A	N/A	N/A	N/A	Yes	Critically low quality
Milas et al.	Yes	No	Yes	Yes	Yes	Yes	Yes	Yes	Yes	No	Yes	Yes	Yes	Yes	Yes	Yes	Low quality
Zehetner et al.	Yes	No	Yes	Yes	Yes	Yes	Yes	Yes	Yes	No	Yes	Yes	Yes	Yes	No	No	Critically low quality
Arezzo et al.	Yes	Yes	Yes	Yes	Yes	Yes	Yes	Yes	Yes	Yes	Yes	Yes	Yes	Yes	Yes	Yes	High quality
Wang et al.	No	No	Yes	Yes	Yes	No	Yes	Yes	No	No	Yes	No	No	Yes	No	No	Critically low quality
Sajid et al.	Partial yes	No	Partial yes	Yes	No	Yes	Yes	Yes	Yes	No	Yes	No	Partial yes	Yes	No	Yes	Critically low quality
Trastulli et al.	Partial yes	No	Yes	Yes	Yes	Yes	Yes	Yes	Yes	No	Yes	Yes	Yes	Yes	Yes	Yes	Low quality
Markar et al.	No	No	Yes	Yes	Yes	No	Yes	Yes	Yes	Yes	Yes	Yes	Yes	Yes	Yes	Yes	Low quality

## Data Availability

The data used to support the findings of this study are available from the corresponding author upon request.
